# snRNA‐seq analysis of human brains suggests cell type‐specific synergistic effects of APOE4 and TREM2‐R47H and potential drug targets in Alzheimer’s Disease

**DOI:** 10.1002/alz.092389

**Published:** 2025-01-03

**Authors:** Diana J Zajac, Jielin Xu, Feixiong Cheng

**Affiliations:** ^1^ Cleveland Clinic, Cleveland, OH USA; ^2^ Case Western Reserve University, Cleveland, OH USA

## Abstract

**Background:**

Alzheimer’s Disease (AD) risk variants *APOE4* and *TREM2‐R47H* have been shown to impact glial cell functions and transcriptional profiles. We hypothesize that *TREM2‐APOE* may have synergistic effects in driving pathogenesis and disease progression of AD in a cell type‐specific manner.

**Methods:**

We investigated cell‐type specific transcriptional changes associated with *APOE4*‐ and *TREM2‐R47H*‐carrier status. We conducted single‐nuclei RNA‐sequencing (snRNA‐seq) analysis, including cell type clustering and annotation, and identification of differentially expressed genes (DEGs). We used integrative genomic analysis for pathway enrichment and drug‐target network inference. Our investigation was between *APOE‐TREM2* groups: *APOE4‐carrier+TREM2‐R47H* (*E4‐R47H*) (n = 18) vs *non‐APOE4+R47H (nonE4‐R47H)* (n = 6), *APOE4‐carrier+TREM2‐common variant* (*E4‐CV)* (n = 16) vs *nonE4‐CV* (n = 6), *E4‐R47H* vs *nonE4‐R47H*, and *E4‐CV* vs *nonE4‐CV*.

**Results:**

We found that *APOE‐TREM2* status had synergistic effects on cell abundance in several cell types, especially in oligodendrocytes, excitatory neurons and pericytes. Pathway enrichment analysis suggests a possible *APOE4*‐ and *TREM2‐R47H*‐dependent regulation of cell type‐specific pathway signaling. Of note, preliminary analysis suggests an *APOE4*‐dependent regulation of *TREM2‐R47H*‐associated PI3K‐Akt signaling in microglia such that *APOE4‐R47H* carriers had significantly upregulated PI3K‐Akt signaling but *nonAPOE4‐R47H* carriers had comparatively downregulated PI3K‐Akt signaling. Oligodendrocyte clusters show *R47H*‐dependent effects of *APOE4*‐carrier status on relevant signaling pathways as well. Oligodendrocytic signaling pathways for long‐term depression and serotonin/anxiety events, as well as sleep regulation and circadian entrainment have *R47H*‐dependent regulation between *APOE4* vs non‐*APOE4* carriers. We employed integrative network‐based analysis of single‐nuclei transcriptomic and drug‐gene signatures and identified an FDA‐approved drug *clidinium bromide*, a treatment for irritable bowel syndrome and enterocolitis, to be a possible antagonist of the synergistic R47H‐APOE4 effects in microglia.

**Conclusion:**

In summary, this study suggests possible synergistic effects of *APOE4*‐carrier and *TREM2‐R47H*‐carrier status on cell type abundance and cell type‐specific specific signaling pathways. Hence, further classification of differences between joint *APOE‐TREM2* genotypes will improve our understanding of glial cell alterations in AD, and could lead to novel microglia‐targeted therapeutic interventions. Such findings enhance our knowledge of druggable therapeutic targets and support the need for precision and personalized medicine. Independent cohort validation and functional observations of candidate targets and treatments are warranted.

